# Physiology and growth of newly bred Basmati rice lines in response to vegetative-stage drought stress

**DOI:** 10.3389/fpls.2023.1172255

**Published:** 2023-05-09

**Authors:** Raheela Waheed, Farah Deeba, Faisal Zulfiqar, Anam Moosa, Muhammad Nafees, Muhammad Ahsan Altaf, Muhammad Arif, Kadambot H. M. Siddique

**Affiliations:** ^1^ National Institute for Biotechnology and Genetic Engineering (NIBGE), Faisalabad, Pakistan; ^2^ Pakistan Institute of Engineering and Applied Sciences (PIEAS), Islamabad, Pakistan; ^3^ International Rice Research Institute, Los Baños, Laguna, Philippines; ^4^ Department of Biochemistry and Biotechnology, The Women University, Multan, Pakistan; ^5^ Department of Horticultural Sciences, Faculty of Agriculture and Environment, The Islamia University of Bahawalpur, Bahawalpur, Pakistan; ^6^ Department of Plant Pathology, Faculty of Agriculture and Environment, The Islamia University of Bahawalpur, Bahawalpur, Pakistan; ^7^ School of Horticulture, Hainan University, Haikou, China; ^8^ The UWA Institute of Agriculture, The University of Western Australia, Perth, WA, Australia

**Keywords:** drought stress, introgressed recombinants, root parameters, root traits, physiological parameter

## Abstract

Basmati rice is inherently sensitive to various environmental stresses. Abrupt changes in climatic patterns and freshwater scarcity are escalating the issues associated with premium-quality rice production. However, few screening studies have selected Basmati rice genotypes suitable for drought-prone areas. This study investigated 19 physio-morphological and growth responses of 15 Super Basmati (SB) introgressed recombinants (SBIRs) and their parents (SB and IR554190-04) under drought stress to elucidate drought-tolerance traits and identify promising lines. After two weeks of drought stress, several physiological and growth performance traits significantly varied between the SBIRs (p ≤ 0.05) and were less affected in the SBIRs and the donor (SB and IR554190-04) than SB. The total drought response indices (TDRI) identified three superior lines (SBIR-153-146-13, SBIR-127-105-12, SBIR-62-79-8) and three on par with the donor and drought-tolerant check (SBIR-17-21-3, SBIR-31-43-4, SBIR-103-98-10) in adapting to drought conditions. Another three lines (SBIR-48-56-5, SBIR-52-60-6, SBIR-58-60-7) had moderate drought tolerance, while six lines (SBIR-7-18-1, SBIR-16-21-2, SBIR-76-83-9, SBIR-118-104-11, SBIR-170-258-14, SBIR-175-369-15) had low drought tolerance. Furthermore, the tolerant lines exhibited mechanisms associated with improved shoot biomass maintenance under drought by adjusting resource allocation to roots and shoots. Hence, the identified tolerant lines could be used as potential donors in drought-tolerant rice breeding programs, administered for subsequent varietal development, and studied to identify the genes underlying drought tolerance. Moreover, this study improved our understanding of the physiological basis of drought tolerance in SBIRs.

## 1 Introduction

Vegetative-stage drought stress decreases global rice production by 21–50.6% due to plant growth reductions of up to 70% (Yang et al., 2008; Lum et al., 2014; Petrozza et al., 2014; Zhang et al., 2018). Furthermore, emerging changes in climate patterns, especially erratic rainfall and temperature fluctuations, will exacerbate drought frequency and duration in the years ahead (Lesk et al., 2016). The rapidly changing climate, declining land resource availability, increased food demands, and increased biotic and abiotic stresses threaten global food security (Gwynn-Jones et al., 2018).

Improvements in drought tolerance require a combination of favorable alleles tagging various traits that promote growth and ultimately maintain yield. Several rice cultivars (e.g., N22, Moroberekan, Aus 276, Kali Aus, and Azucena) have been used as tolerance donors in breeding programs (Kumar et al., 2014) for introgression and pyramiding large-effect quantitative trait loci (QTL) into various elite mega-varieties of rice through marker-assisted breeding schemes. In the present study, drought-tolerant genotype IR55419-04 (Sabar and Arif, 2014; Dixit et al., 2017) was crossed with Super Basmati (SB) to develop SB introgressed recombinant (SBIR) lines using marker-assisted backcrossing to the BC_3_F_7_ generation. The developed SBIRs harbor target QTL—*qOA1* on chromosome 1 for osmotic adjustment by Robin et al. (Robin et al., 2003), *qDRDW4* on chromosome 4 for deep root dry weight by Yue et al. (Yue et al., 2006), and *qRL9* on chromosome 9 for maximum root length by Courtois et al. (Courtois et al., 2000)—for drought tolerance traits in different combinations (**Table 1**).

**Table 1:** Super Basmati introgressed recombinants (SBIRs) with target quantitative trait loci (QTL).

Line no.	Line	Introgressed QTL^a^	Yield under drought QTL^b^
P1 (donor)	IR55419-04	*qOA1+qDRDW4+qRL9*	
P2 (recurrent)	Super Basmati	0	
1	SBIR-7-18-1	*qOA1+qDRDW4+qRL9*	
2	SBIR-16-21-2	*qDRDW4+qRL9*	
3	SBIR-17-21-3	*qOA1+qDRDW4+qRL9*	
4	SBIR-31-43-4	*qOA1+qDRDW4+qRL9*	
5	SBIR-48-56-5	*qDRDW4+qRL9*	
6	SBIR-52-60-6	*qOA1+qRL9*	
7	SBIR-58-60-7	*qRL9*	
8	SBIR-62-79-8	*qOA1+qDRDW4+qRL9*	*qDTY1.1+qDTY1.2+qDTY2.2+qDTY3.1*
9	SBIR-76-83-9	*qOA1+qRL9*	
10	SBIR-103-98-10	*qOA1+qDRDW4+qRL9*	
11	SBIR-118-104-11	*qDRDW4+qRL9*	
12	SBIR-127-105-12	*qRL9*	
13	SBIR-153-146-13	*qOA1+qDRDW4+qRL9*	*qDTY3.1+qDTY3.2+qDTY6.1*
14	SBIR-170-258-14	*qOA1+qDRDW4+qRL9*	*qDTY3.1+qDTY3.2*
15	SBIR-175-369-15	*qOA1+qDRDW4*	

a Target QTL: qOA1 = QTL on chromosome 1 for osmotic adjustment by Robin et al. (Robin et al., 2003); qDRDW4 = QTL on chromosome 4 for deep root dry weight by Yue et al. (Yue et al., 2006); qRL9 = QTL on chromosome 9 for maximum root length by Courtois et al. (Courtois et al., 2000).

b Other than introgressed QTL: qDTY = QTL for yield under drought, number indicates chromosome number.

The impact of drought on plants is complex and influenced by environmental conditions. Under drought stress, selection based on yield is challenging due to its polygenic nature (Turner et al., 2014) but can be aided by measuring growth and physiological traits indicative of the environment × adaptability interaction (Dolferus, 2014). Superior physiological and growth responses under vegetative-stage drought stress may be associated with improved yield under reproductive-stage drought stress (Pantuwan et al., 2002) and can be early indicators for selecting drought-tolerant genotypes. However, few screening studies have selected Basmati rice genotypes suitable for drought-prone areas. To date, there are no drought-tolerant Basmati rice cultivars available for rice farmers to combat the effects of drought stress in Pakistan. Therefore, we aimed to complement ongoing work on improving the drought tolerance of SB rice (Sabar and Arif, 2014; Sabar et al., 2019) by characterizing vegetative-stage drought response traits in a set of SBIR lines.

Rice is the staple food in Asia, and the current climate scenario threatens its production. An estimated 26% increase in rice production is needed by 2035 to feed the growing population (Seck et al., 2012). Premium-quality Basmati rice represents the heritage and pride of Pakistan, where it is typically cultivated under flooded conditions through irrigation. Pakistan’s Kallar tract zone is renowned for producing Basmati rice varieties with excellent aroma, super fine grain, and good cooking and eating qualities (Pawan et al., 2012). Despite the high demand for this special Basmati rice, few attempts have been made to protect its heritage or boost its production to increase its economic sustainability. Declining water resources are the primary constraint to Pakistan’s rice productivity system, resulting in periodic drought stress (Mehmood et al., 2020).

We undertook a vegetative-stage drought experiment using SBIR lines with selected QTL combinations to (i) dissect the physiological basis of superior lines previously identified for drought tolerance under field conditions, (ii) identify relationships among physiological parameters, and (iii) identify promising drought-tolerant SBIRs compared to check and parental lines for advancing the varietal release pipeline and thus contributing to sustainable future strategies for Basmati rice production.

## 2 Results

### 2.1 Phenotypic performance under drought stress

All recombinants had wide ranges of genetic variation for the measured traits, with coefficients of variation (CV) ranging from 7.92–58.4%. CRPT, %R_SDW, LOP, RGR, RDW, CC had the highest CV (>30%), while ten traits had medium CV (WUE, TN, PH, LGA, SDW, RSR, CRN, RL, RTi, RF) and four traits (WU, RAD, MRL, and RSR) had the lowest CV (<15%). The WS treatment significantly reduced TN, LGA, RGR, PH, SDW, RDW, RSR, CRN, and RAD but enhanced RL, RTi, and RF in the SBIR seedlings relative to the WW treatment. Significant variation (p< 0.05) existed for TN, CRN, and RF among the tested lines under drought stress, with highly significant variation (p< 0.01) for CC, RTi, and CRPT. No significant variations (p *‗* 0.05) occurred for LGA, RGR, LOP, RDW, RSR, MRL, RL, or RAD among the tested lines under WS or WW conditions (**Table 2**).

**Table 2:** Descriptive statistics for all studied traits under well-watered (WW) and water-stressed (WS) conditions.

Variable		Min	Max	Mean ± SE	CV (%)	G
**%R_SDW**	WW	–	–	–	–	–
	WS	-51.05	64.2	38.95± 3.49	53.83	ns
**WU**	WW	–	–	–	–	–
	WS	0.34	0.48	0.42 ± 0.005	7.57	ns
**WUE**	WW	–	–	–	–	–
	WS	1.37	3.87	1.92 ± 0.068	21.2	ns
**TN**	WW	3	10	6.19 ± 0.27	26.68	ns
	WS	3	7	4.2 ± 0.17	24.1	*
**PH**	WW	35.1	57.35	44.6 ± 1.08	14.5	***
	WS	13.05	49.84	29.26 ± 1.5	30.8	ns
**LGA**	WW	181.7	361	269.4 ± 8.5	19.1	ns
	WS	40.8	140.3	82.6 ± 3.8	27.53	ns
**RGR**	WW	0.101	0.17	0.139 ± 0.002	11.76	ns
	WS	0.013	0.19	0.093 ± 0.007	46.9	ns
**CC**	WW	1.35	12.9	6.27 ± 0.48	46.2	ns
	WS	1.7	12.0	4.1 ± 0.44	58.4	**
**LOP**	WW	–1.51	–0.76	–1.31 ± 0.02	10.87	ns
	WS	–8.4	–1.15	–2.62 ± 0.2	48.6	ns
**SDW**	WW	0.879	2.085	1.39 ± 0.04	19.19	**
	WS	0.46	1.78	0.819 ± 0.04	26.37	ns
**RDW**	WW	0.17	0.66	0.426 ± 0.02	29.6	ns
	WS	0.062	0.72	0.204 ± 0.016	48.5	ns
**RSR**	WW	0.165	0.53	0.31 ± 0.014	27.9	ns
	WS	0.13	0.4	0.24 ± 0.009	23.2	ns
**CRN**	WW	33	110	73.1 ± 2.57	21.15	*
	WS	21	89	39.0 ± 1.9	29.2	*
**MRL**	WW	20.2	30	24.8 ± 0.47	11.4	ns
	WS	18	35	24.4 ± 0.63	15.51	ns
**CRPT**	WW	8.7	25	12.74 ± 0.57	27.01	**
	WS	5.1	29.6	9.64 ± 0.7	44.4	**
**RL**	WW	798.7	1994.4	1246 ± 46.8	22.5	ns
	WS	822.73	2319.3	1694 ± 69.2	24.5	ns
**RAD**	WW	0.175	0.26	0.20 ± 0.003	10.18	ns
	WS	0.13	0.22	0.178 ± 0.003	13.3	ns
**RTi**	WW	5672	17559	10784 ± 498.2	27.72	ns
	WS	5114	23534	12763 ± 676.9	31.8	**
**RF**	WW	4660	16343	8807.1 ± 469	31.97	*
	WS	7868	28812	18790 ± 933	29.8	*
**%LR**	WW	69.69	86.46	77.4 ± 0.6	4.49	**
	WS	78.2	88.04	83.2 ± 0.4	2.6	ns

Min, minimum; Max, maximum; SE, standard error; CV, coefficient of variation; G, genotypes varied significantly at * p< 0.05, ** p< 0.01, *** p< 0.001. ns, non-significant; % R_SDW, % reduction in shoot dry weight; WU, water uptake (kg); WUE, water use efficiency (g L^–1^); TN, tiller number; CC, chlorophyll content; (µmol m^–2^) PH, plant height (cm); LGA, leaf green area (cm^2^); RGR, relative growth rate; LOP, leaf osmotic potential (MPa); SDW, shoot dry weight (g); RDW, root dry weight (g); RSR, root: shoot dry weight ratio; CRN, crown root number; MRL, maximum root length (cm); CRPT, crown root per tiller; RL, root length (cm); RAD, root average diameter (mm); RTi, root tips; RF, root forks; %LR, percent lateral roots. ‘–’ indicates missing values.

### 2.2 Total drought response index

The TDRI of the SBIRs ranged from 13.6–18.73, with six lines (SBIR-7-18-1, SBIR-16-21-2, SBIR-76-83-9, SBIR-118-104-11, SBIR-170-258-14, SBIR-175-369-15) identified with low drought tolerance (13.5–15), three lines (SBIR-48-56-5, SBIR-52-60-6, SBIR-58-60-7) as moderately tolerant (15.1–17), and six lines (SBIR-17-21-3, SBIR-31-43-4, SBIR-62-79-8, SBIR-103-98-10, SBIR-127-105-12, SBIR-153-146-13) as highly drought tolerant (>17.1). Moreover, the highly drought-tolerant lines were superior to both parents (donor IR55419-04, recurrent Super Basmati) and the drought-tolerant check variety (Azucena) (**Table 3**).

**Table 3:** Grouping of Super Basmati introgressed recombinants (SBIRs) based on the total drought response index (TDRI) calculated from shoot and root growth parameters*.

Classification	Lines	TDRI
Low tolerance	SBIR-7-18-1	14.08513
13.5–15	SBIR-16-21-2	14.12541
	SBIR-76-83-9	13.95742
	SBIR-118-104-11	13.63789
	SBIR-170-258-14	14.70197
	SBIR-175-369-15	14.5022
Moderate tolerance	SBIR-48-56-5	16.82162
15.1–17	SBIR-52-60-6	15.7308
	SBIR-58-60-7	15.41342
Tolerant	SBIR-17-21-3	17.87805
17–19	SBIR-31-43-4	17.90453
	SBIR-62-79-8	18.23459
	SBIR-103-98-10	17.67977
	SBIR-127-105-12	18.30323
	SBIR-153-146-13	18.73186
P1	IR-554190	16.48336
P2	Super Basmati	12.52044
Check	Azucena	17.6518

P1: Parent 1 (donor), P2: Parent 2 (recipient, recurrent), Check: drought-tolerant check.

*Tiller number, chlorophyll content (µmol m^–2^), plant height (cm), leaf green area (cm^2^), relative growth rate, leaf osmotic potential (MPa), shoot dry weight (g), root dry weight (g), root: shoot dry weight ratio, crown root number, maximum root length (cm), crown roots per tiller, root length (cm), average root diameter (mm), root tips, root forks, and % lateral root.

### 2.3 Physio-morphological responses of shoots

**Figure 1:**
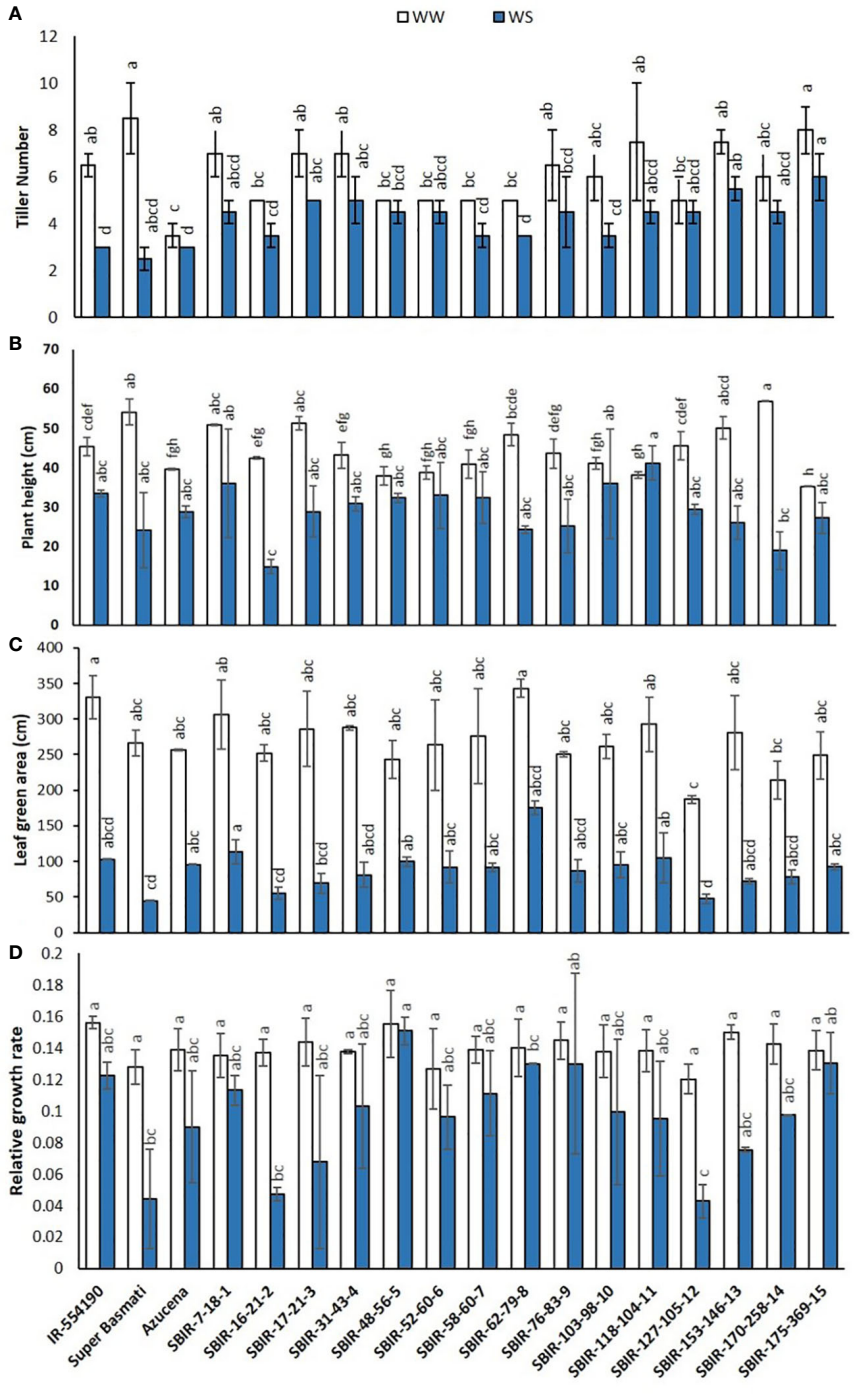
Shoot parameter responses of selected Super Basmati introgressed recombinants (SBIRs) under well-watered (WW) and water-stressed (WS) conditions: **(A)** tiller number, **(B)** plant height (cm), **(C)** leaf green area (cm^2^), and **(D)** relative growth rate. Vertical bars represent mean ± S.E. The WW and WS treatments for each line were compared for significance at p< 0.05; mean bars with different letters significantly differ.

**Figure 2:**
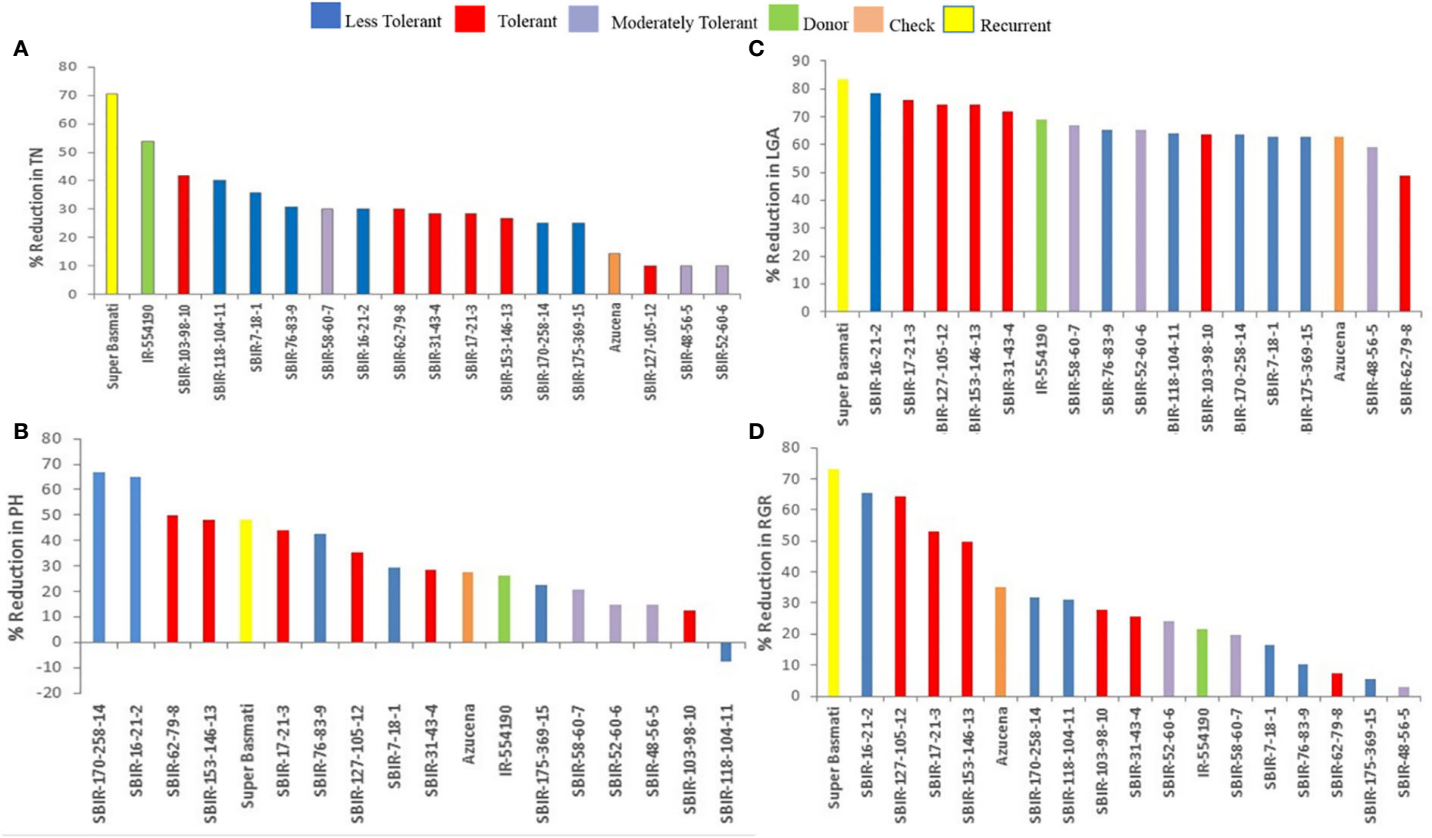
Effect of water stress on **(A)** tiller number, **(B)** plant height (cm), **(C)** leaf green area (cm^2^), and **(D)** relative growth rate of selected lines. Bar colors indicate the drought-tolerant group as determined by TDRI.

Of the measured shoot growth traits, the WS treatment mainly affected TN, PH, LGA, and RGR. SBIR-175-369-15 produced the maximum TN under WW and WS conditions, while SBIR-62-79-8 produced the lowest TN under WS, which significantly differed from the other SBIRs (**Figure 1A**). SBIR-103-98-10 had the highest relative reduction in TN in the WS treatment (41.6%), while SBIR-48-56-5, SBIR-52-60-6, and SBIR-127-105-12 had the lowest (all 10%). Overall, the SBIRs had lower relative reductions in TN than both parents and the tolerant check (**Figure 2A**). The WS treatment substantially decreased PH of most SBIRs (**Figure 1B**), with SBIR-170-258-14 and SBIR-16-21-2 declining the most, while SBIR-118-104-11 did not change (**Figure 2B**).

Leaf growth in rice reflects cell division and expansion, with significant changes in LGA observed under drought stress. Tolerant genotype SBIR-62-79-8 had the greatest LGA (175.42 cm^2^), while SBIR-127-105-12 (also tolerant) had the lowest (47.68 cm^2^) (**Figure 1C**). Overall, all SBIRs had higher LGA than recurrent parent SB under WS. SBIR-16-21-2 had the greatest relative LGA % reduction in the WS treatment, compared to WW, followed by SBIR-62-79-8, while SBIR-48-56-5 had the lowest reduction (**Figure 2C**).

The SBIRs had higher RGRs than the recurrent parent SB, ranging from 0.044 (SBIR-127-105-12) to 0.15 (SBIR-48-56-5). Four lines (SBIR-48-56-5, SBIR-62-79-8, SBIR-76-83-9, SBIR-175-369-15) had higher RGRs than both parents and the tolerant check Azucena (**Figure 1D**). The WS treatment reduced RGR the least for SBIR-16-21-2 (65.3%), followed by SBIR-127-105-12 (64.4%) and SBIR-62-79-8 (64.4%) (**Figure 2D**).

**Figure 3:**
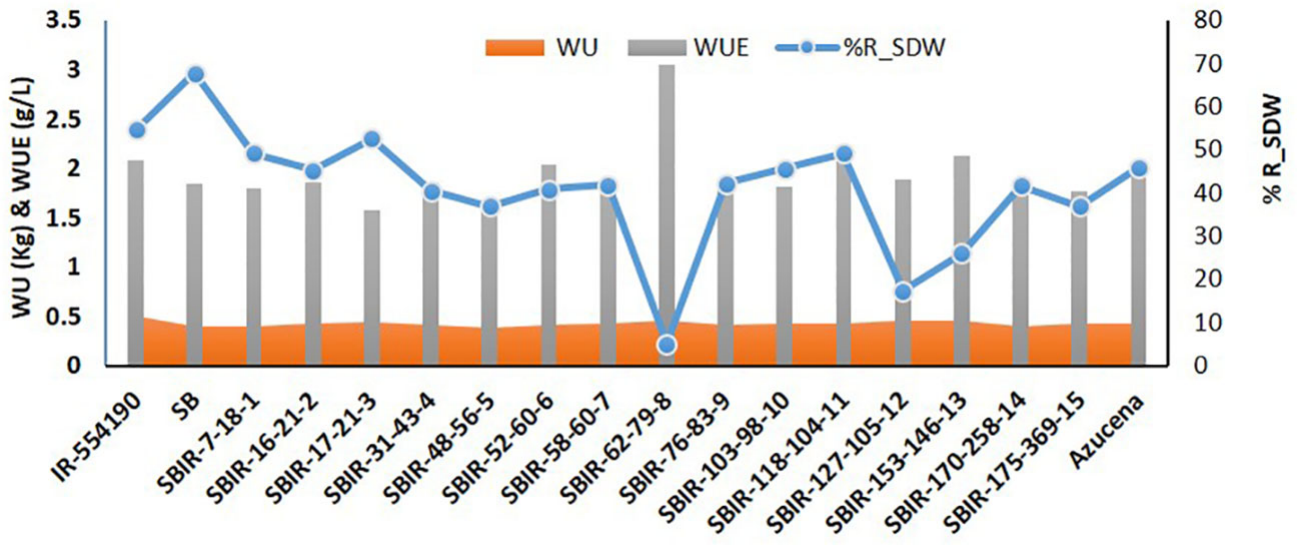
Comparison of water uptake (WU), water use efficiency (WUE), and percent reduction in shoot dry weight (R_SDW) of selected Super Basmati introgressed recombinants (SBIRs).

Physiological analysis of the SBIRs revealed differences in their responses to water stress. The parental lines (IR55419-04 and SB) had WUE values of 2.08 g L^–1^ and 1.84 g L^–1^, respectively, while the SBIRs ranged from 1.57–3.04 g L^–1^. SBIR-62-79-8 (tolerant genotype) had the highest WUE, greater than both parents and the check, while tolerant genotype SBIR-17-21-3 and moderately tolerant genotype SBIR-48-56-5 had the lowest values. Overall, nine SBIRs had lower WUE than both parents (**Figure 3**).

**Figure 4:**
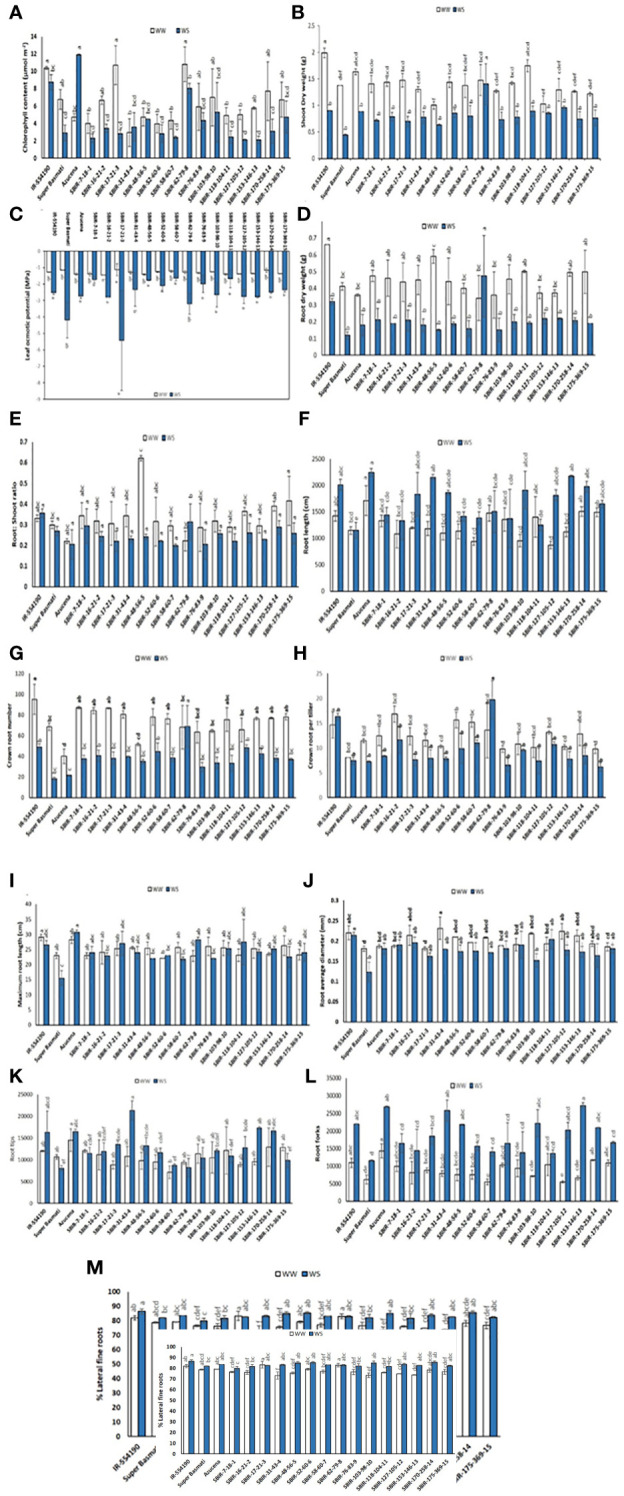
Variation in selected Super Basmati introgressed recombinants (SBIRs) under well-watered (WW) and water-stressed (WS) conditions: **(A)** chlorophyll content (µmol m^–2^), **(B)** shoot dry weight (g), **(C)** leaf osmotic potential (MPa), **(D)** root dry weight (g), **(E)** root: shoot dry weight ratio, **(F)** root length (cm), **(G)** crown root number, **(H)** crown roots per tiller, **(I)** maximum root length (cm), **(J)** root average diameter (mm), **(K)** root tips, **(L)** root forks, and **(M)** percent lateral roots. Vertical bars represent mean ± S.E. The WW and WS treatments for each line were compared for significance at p< 0.05; mean bars with different letters significantly differ.

**Figure 5:**
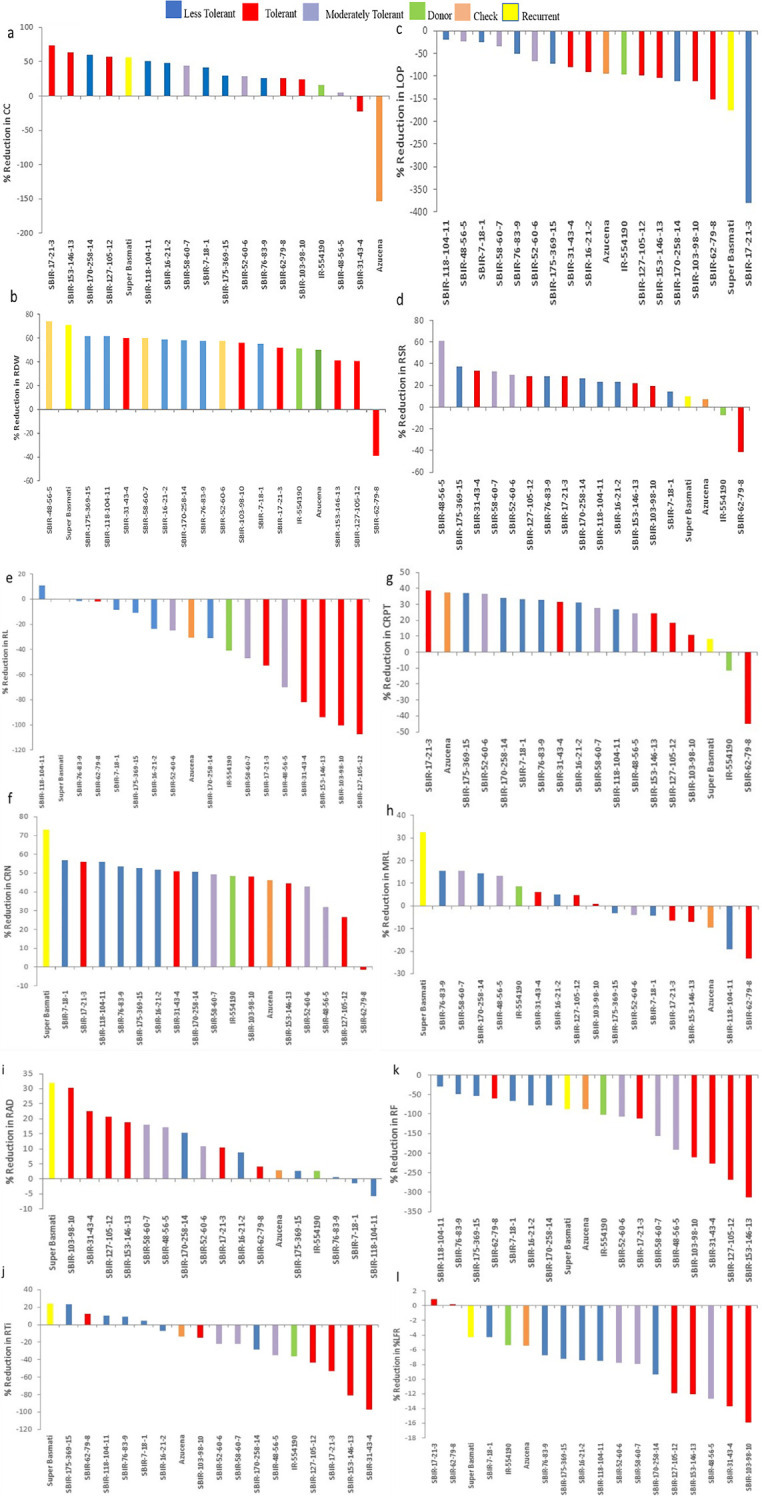
Effect of water stress on **(A)** chlorophyll content (µmol m^–2^), **(B)** root dry weight (g), **(C)** leaf osmotic potential (MPa), **(D)** root: shoot dry weight ratio, **(E)** root length (cm), **(F)** crown root number, **(G)** crown roots per tiller, **(H)** maximum root length (cm), **(I)** root average diameter (mm), **(J)** root tips, **(K)** root forks, and **(L)** percent lateral roots of selected lines. Bar colors indicate the drought-tolerant group as determined by TDRI.

Significant variation in CC occurred among tested lines (p< 0.01). Under WS, eight lines had higher CC values than the recurrent parent SB, with SBIR-62-79-8 the highest (8.025 µmol m^–2^), significantly different from the other SBIRs but closer to the tolerant check, Azucena (11.9 µmol m^–2^) (**Figure 4A**). However, SBIR-17-21-3 had the greatest relative reduction in CC (73.7%), followed by SBIR-153-146-13 (63.3%), SBIR-170-258-14 (59.8%), and SBIR-127-105-12 (57.5%) under WS. Apart from these four lines, the remaining SBIRs performed well with lower reductions in CC than the susceptible recurrent parent SB. Interestingly, the WS treatment increased CC by 22% in SBIR-31-43-4 and 153% in Azucena (tolerant check) relative to WW conditions (**Figure 5A**).

Under WS conditions, all lines had greater SDW than the recurrent parent Super Basmati. However, the tolerant genotype SBIR-62-79-8 had the highest value (1.4 g), which differed significantly from the other SBIRs (**Figure 4B**). The WS treatment significantly reduced SDW (>45%) in five genotypes (SBIR-7-18-1, SBIR-16-21-2, SBIR-17-21-3, SBIR-103-98-10, SBIR-118-104-11), with the lowest reduction in SBIR-62-79-8 (5%), followed by SBIR-127-105-12 (17.3%) and SBIR-153-146-13 (25.9%).

The LOP of SBIRs ranged from –5.4 to –1.65 MPa under WS. SBIR-17-21-3 had the lowest leaf osmotic potential at –5.4 MPa, while SBIR-58-60-7 had the highest (–1.65 MPa), followed by SBIR-118-104-11 (–1.69 MPa), SBIR-7-18-1 (–1.74 MPa), SBIR-48-56-5 (–1.77 MPa), SBIR-76-83-9 (–1.99 MPa) and SBIR-52-60-6 (–2.1 MPa). Nine SBIRs had higher LOP values than the donor and recurrent parents and the tolerant check (**Figure 4C**). Mean LOP significantly declined (α< 0.05) under WS in all tested lines (**Figure 5C**).

### 2.4 Root responses

Significant differences were observed for RDW, CRN, CRP, TRTi, RF, and %LR under WS. Overall, SBIR-62-79-8 had the highest RDW (0.47 g), followed by SBIR-127-105-12 (0.22 g), while SBIR-48-56-5 had the lowest value (0.15 g) (**Figure 4D**). Across all tested lines, the WS treatment significantly reduced RDW, except in SBIR-62-79-8, which increased by 38.8% relative to WW conditions (**Figure 5B**). SBIR-62-79-8 had the highest RSR (0.31), followed by SBIR-7-18-1 (0.29) and SBIR-170-258-14 (0.28), all of which were higher than the recurrent parent SB. The remaining 13 lines had lower RSR values than both parents with non-significant differences (LSD test, α = 0.05) (**Figures 4**, **5D**). SBIR-48-56-5 had the greatest reduction in RSR (61.1%), while SBIR-62-79-8 increased relative to the control. The WS treatment increased RL in all lines relative to WW conditions, except for SBIR-118-104-11, with RL ranging from 1,252.4 cm (SBIR-118-104-11) to 2,154.1 cm (SBIR-31-43-4) (**Figure 4F**). Overall, the WS treatment increased RL in all SBIRs more than the recurrent parent SB. Moreover, two lines, SBIR-31-43-4 and SBIR-153-146-13, had longer roots than the donor parent, IR55419-04. Azucena, the tolerant check, had the greatest RL among all tested genotypes (**Figure 5E**). All tested lines had greater CRN and CRPT than the recurrent parent SB (**Figures 4G**, **5H**). However, only one line, SBIR-62-79-8, had greater CRN than the donor parent and drought-tolerant check, with a 1.5% increase under drought stress (**Figure 5F**).

The MRL of SBIRs ranged from 28.15 cm (SBIR-62-79-8) to 21.75 cm (SBIR-58-60-7). The WS treatment increased MRL in seven lines (SBIR-7-18-1, SBIR-17-21-3, SBIR-52-60-6, SBIR-62-79-8, SBIR-118-104-11, SBIR-153-146-13, SBIR-175-369-15) relative to WW conditions (**Figure 4I**). Overall, all SBIRs improved MRL relative to SB. In addition, three lines from the tolerant group (SBIR-62-79-8, SBIR-17-21-3, SBIR-118-104-11) had greater MRL than the donor parent IR55419-04 (**Figure 5H**). All SBIRs had higher RAD than the recurrent parent SB but lower RAD than the donor parent IR55419-04. The RAD ranged from 0.152 mm (SBIR-103-98-10) to 0.2 mm (SBIR-118-104-11) (**Figure 4J**). Water stress decreased the RAD of all lines relative to WW conditions, except SBIR-118-104-11 and SBIR-7-18-1, with 5.6% and 1.4% increases, respectively (**Figure 5I**).

Significant differences occurred among the SBIRs for RTi, RF, and %LR, with superior performance to SB. SBIR-31-43-4 had the highest RTi (21,324), while SBIR-62-79-8 had the lowest (8,237) (**Figure 4K**) and the greatest reduction (23.1%) under drought stress (**Figure 5J**). Overall, the number of RTi under WS conditions increased for ten lines relative to WW conditions (**Figure 4K**). The WS treatment significantly increased RF in all lines relative to SB (**Figure 5L**). Except for SBIR-62-79-8 and SBIR-17-21-3, all SBIRs increased their %LR under stress relative to the control (**Figures 4M**, **5L**).

### 2.5 Correlation

**Figure 6:**
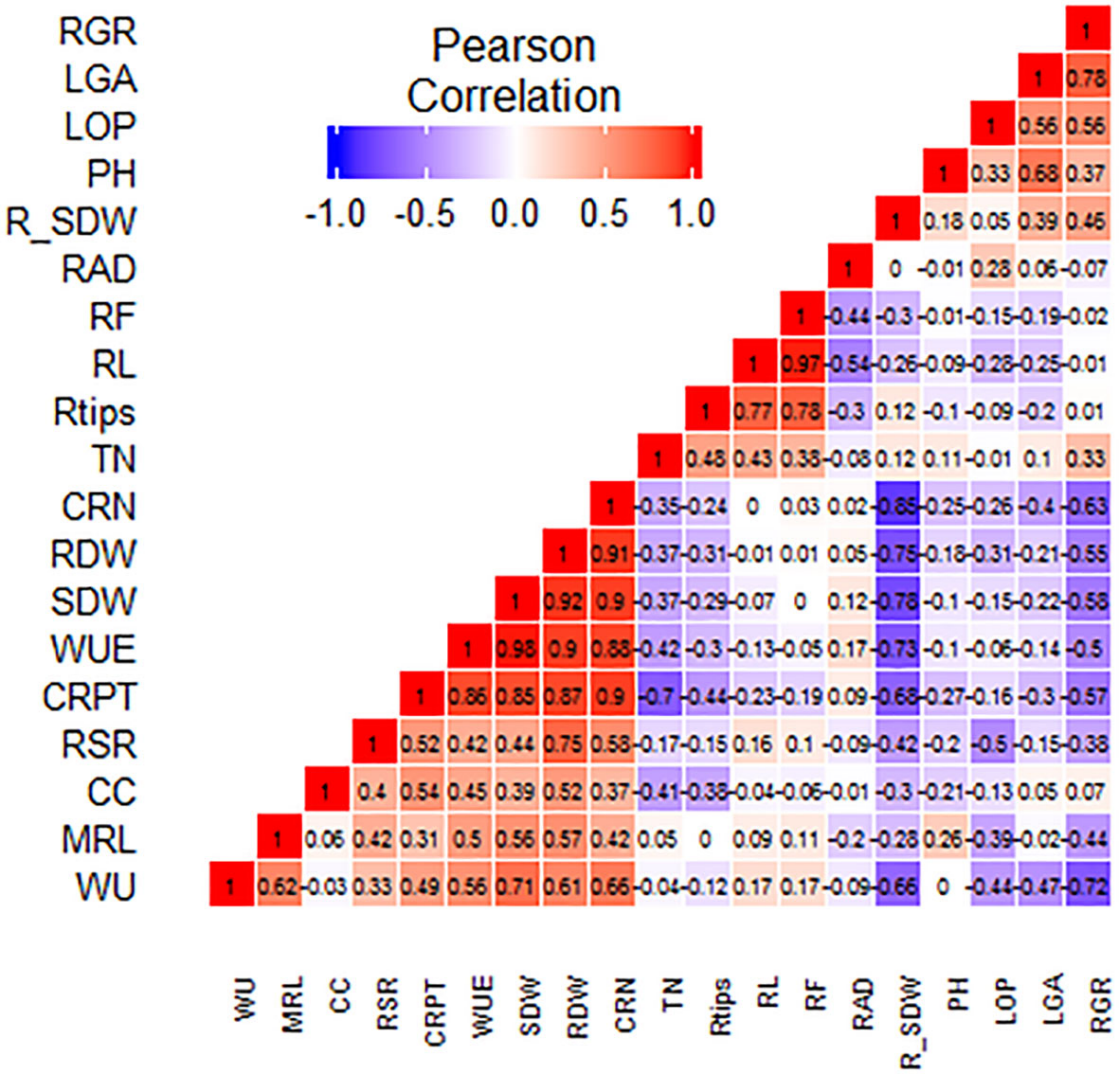
Graphical correlation matrices of the studied traits, with positive correlations shown in red and negative correlations shown in blue. WU: water uptake (kg), MRL: maximum root length (cm), CC: chlorophyll content (µmol m^–2^), RSR: root: shoot dry weight ratio, CRPT: crown root per tiller, WUE: water use efficiency (g L^–1^), SDW: shoot dry weight (g), RDW: root dry weight (g), CRN: crown root number, TN: tiller number, RTi: root tips, RL: root length (cm), RF: root forks, RAD: root average diameter (mm), R_SDW: % reduction in shoot dry weight, PH: plant height (cm), LOP: leaf osmotic potential (MPa), LGA: leaf green area (cm^2^), RGR: relative growth rate.

Under drought stress at the vegetative stage, the percent reduction in SDW (%R_SDW) had a highly significant (p ≤ 0.01) negative correlation with WU, SDW, WUE, RDW, CRN, and CRPT. WU of the SBIRs positively correlated (p ≤ 0.05) with WUE and CRPT. WU also had highly significant positive correlations with RDW, SDW, MRL, and CRN and a negative correlation with RGR. Moreover, WUE had a significant negative correlation with RGR but highly significant (p ≤ 0.05) positive correlations with SDW, CRN, CRPT, RDW, and MRL. The CC of the SBIRs had significant (p ≤ 0.05) positive correlations with RDW and CRPT. LOP, LGA, and RGR positively correlated with each other. PH had a highly significant positive association with LGA, while LOP negatively correlated with RSR. RDW positively correlated with SDW, with both traits having highly significant (p ≤ 0.05) positive correlations with WU, WUE, and root traits (CRN, CRPT, MRL) but negative correlations with RGR. Additionally, RGR had significant negative correlations with CRPT and CRN, while CRN, CRPT, and RSR positively (p ≤ 0.05) correlated with each other. RL had highly significant positive correlations with RTi and RF, while RAD had a highly significant (p ≤ 0.01) positive correlation with RF but a negative correlation with RL (**Figure 6**).

## 3 Discussion

Few studies have investigated improving Basmati rice in drought-prone areas. No drought-tolerant Basmati rice cultivar is available for rice farmers to combat the impact of drought stress under Pakistan’s current climatic and water resource situation. Therefore, improving the drought tolerance of Basmati rice is vital to meet future challenges associated with its sustainable production. This study investigated the impact of early vegetative-stage water stress on Basmati rice to dissect the physiological basis of superior lines previously identified for drought tolerance under field conditions.

Drought decreases plant performance by obstructing water provision to roots and increasing transpiration rates (Mahajan and Tuteja, 2005; Shao et al., 2009). In addition, water scarcity reduces plant water potential, affecting physio-metabolic pathways and decelerating plant growth rates (Verslues et al., 2006). In all studied lines, drought stress decreased TN, LGA, RGR, LOP, CC (except SBIR-31-43-4), SDW, CRN, PH (except SBIR-118-104-11), RDW (except SBIR-62-79-8), RSR (except SBIR-62-79-8), and CRN (except SBIR-62-79-8) and increased RL (except SBIR-62-79-8), RTi (except SBIR-62-79-8), RF (except SBIR-62-79-8), and %LR (except SBIR-62-79-8, SBIR-17-21-3), consistent with others (Ying et al., 2015). Our study also revealed that three traits—LOP, LGA, and RGR—positively correlated with each other under WS (**Figure 6**), as reported by Babu et al. (Babu et al., 1999).

Unlike the IRRI standard evaluation system of drought tolerance (sensitivity and recovery scores; IRRI 2014), the TDRI is based on the total variability of traits measured and can serve as selection criteria in stress-tolerant breeding programs (Wijewardana et al., 2015; Singh et al., 2017). As a result, we identified six drought-tolerant SBIRs (SBIR-17-21-3, SBIR-31-43-4, SBIR-62-79-8, SBIR-103-98-10, SBIR-127-105-12, SBIR-153-146-13) that could be used in drought-prone areas together with water-saving strategies for improving commercial rice production. These six lines had higher TDRI values than the recurrent parent ‘SB,’ drought-tolerant donor ‘IR55419,’ and drought-tolerant check ‘Azucena’ (**Table 3**). Moreover, the TDRI tolerance indices of each trait revealed that different lines had different tolerance mechanisms. SBIR-153-146-13 and SBIR-127-105-12 ranked first and second among all tested lines (**Table 3**). Notably, the RL of SBIR-127-105-12 increased by 107.2% under drought stress. The CC, RAD, and RGR did not appear to contribute to the drought tolerance of these high-performing lines but rather the superior root architecture, especially lateral root growth (Rti, RF, and %LR) that favors WUE, thus regulating water balance to increase SDW (**Table 4**). Studies by Courtois et al. (Courtois et al., 2000), Yu et al. (Yu et al., 2007), Kanbar et al. (Kanbar et al., 2009), and Nguyen et al. (Nguyen et al., 2009) also reported that plant WUE directly depends on root-related traits. Furthermore, studies have shown that higher RSR increases soil resource attainment and favors plant adaptation to drought (Asch et al., 2005; Prasad et al., 2008; Xu et al., 2015; Sehgal et al., 2018). Moreover, a continuous increase in young Rti is vital for drought-tolerant plants to uptake water and nutrients from the soil (Robinson et al., 1991). Studies have shown that increased root biomass supports aboveground biomass production in drought-tolerant rice under drought stress (Bengough et al., 2011; Kanjoo et al., 2012; Liu et al., 2014; Ju et al., 2015; Chu et al., 2016). In this study, rice subjected to drought stress during early growth increased root mass by relocating assimilates to roots, improving aboveground growth. Increased Rti, RF, and %LR improved the ability of roots to penetrate deeper soil, contributing to the drought avoidance mechanism in rice (Uga et al., 2008). It was evident that the prolific RL, Rti, RF, and %LR were effective for drought tolerance of all lines except SBIR-62-79-8.

**Table 4:** Groupings of traits (high, medium, or low values) related to the performance of drought-tolerant lines based on the total drought response index (TDRI).

SBIR-153-146-13			SBIR-127-105-12			SBIR-62-79-8			SBIR-17-21-3			SBIR-31-43-4			SBIR-103-98-10		
High	Medium	Low	High	Medium	Low	High	Medium	Low	High	Medium	Low	High	Medium	Low	High	Medium	Low
SDW CRPT RL RTi RF %LR R_SDW	TN RDW LOP RSR CRN MRL WUE	CC LGA RGR RAD PH	TN SDW CRN CRPT RL RTi RF %LR R_SDW	RDW LOP PH MRL	CC RGR LGA RAD	WUE LGA RGR CC SDW RDW RSR CRN CRPT MRL RAD R_SDW	TN RF LOP	PH RL RTi RF %LR	LOP MRL RL RTi	TN RDW RSR RAD RF R_SDW	CC WUE SDW LGA PH RGR CRN CRPT %LR %R_SDW	CC RL RTi RF %LR	TN RDW SDW LOP PH RGR RSR CRN CRPT LGA %R_SDW	MRL RAD	CC PH CRPT RL RF %LR	RDW LGA LOP RGR RSR CRN MRL RTi %R_SDW	TN RAD

% R_SDW, % reduction in shoot dry weight; WUE, water use efficiency (g L^–1^); TN, tiller number; CC, chlorophyll content; (µmol m^–2^), PH, plant height (cm); LGA, leaf green area (cm^2^); RGR, relative growth rate; LOP, leaf osmotic potential (MPa); SDW, shoot dry weight (g); RDW, root dry weight (g); RSR, root shoot dry weight ratio; CRN, crown root number; MR:, maximum root length (cm); CRPT, crown root per tiller; RL, root length (cm); RAD, root average diameter (mm); RTi, root tips; RF, root forks; %LR, percent lateral roots.

SBIR-62-79-8 is a high-performing drought-tolerant line with distinct physiological mechanisms compared to the other drought-tolerant SBIRs under WS. It had numerous superior traits (CC, WUE, LGA, RGR, SDW, RDW, RSR, CRN, CRPT, MRL, and RAD) and the lowest reduction in SDW under WS, but unlike the other tolerant lines, its RL, Rti, and %LR did not increase under WS, indicating a role of nodal roots rather than lateral roots in its drought tolerance.

SBIR-31-43-4 stood out for CC under WS, increasing by 22% relative to WW conditions (**Figure 5A**), which could be due to increased antioxidant enzyme activities (Nahakpam, 2017) or the stay-green effect as physiological adaptations to drought. Another line, SBIR-103-98-10, had a minimal reduction in CC. SBIR-31-43-4 and SBIR-103-98-10 had incremental changes in RL, Rti, RF, and %LR under WS but were inferior in RAD. The drought tolerance route in these lines appears related to high CC, increasing photosynthesis and productivity. Other studies have reported increased CC under abiotic stress, interpreted as a plant survival strategy through avoidance (Alaei, 2011; Khayatnezhad et al., 2011). In line with this, the stable CC of tolerant lines (except SBIR-17-21-3) under WS (**Figures 3**, **4A**) was associated with the physiological ability to mitigate drought effects.

Our results revealed that SBIR-17-21-3 increased soil water extraction through its MRL but had the highest reductions in WUE, SDW, and CC under WS, suggesting WUE and CC maintenance do not confer the drought tolerance mechanism as in the other tolerant lines. In contrast, it could be attributed to a higher LOP and favorable root architecture, as observed in the small decline in RDW, RSR, RL, MRL, Rti, and RF under WS (**Table 4**). Moreover, the lower CC of this line under WS might be due to the increased production of reactive oxygen species impairing chlorophyll synthesis and deteriorating the photosynthetic apparatus or its membranes. The reduced shoot growth might be due to the decline in photosynthesis and cell expansion (Jaleel et al., 2008).

Likewise, Borras et al. (Borrás et al., 2004) reported that drought stress decreased the photosynthetic rate, with its variability correlated with grain yield under water deficit conditions.

In our study, moderately tolerant lines (SBIR-48-56-5, SBIR-52-60-6, SBIR-58-60-7) exhibited inhibitory effects of drought stress, with the lowest reductions in PH, TN, LGA, and RGR. In SBIR-48-56-5, the WS treatment significantly decreased WUE, RDW, RSR, and LOP but maintained SDW (mean reduction of 37%), CC (5% reduction), CRN (32% reduction), CRPT (24% reduction), RL (70% increase), RAD (17.2%), Rti (35.2% increase), RF (191% increase), and %LR (12.6%) (**Figures 3**, **5**), suggesting that this line can maintain photosynthesis and shoot biomass under drought stress. In contrast, SBIR-52-60-6 and SBIR-58-60-7 had mild reductions in the studied attributes, with increases in MRL, RL, Rti, RF, and %LR (**Figure 5**). The overall findings indicate different routes to drought tolerance, conferred by different combinations of traits, even among lines with the same parents (**Table 4**). Moreover, among the drought indicators, stability in WUE, CC, and SDW is crucial for drought tolerance; hence, improving these traits should be targeted for sustainable agriculture (Yang et al., 2019).

The results of this study suggest that the improved drought tolerance of newly bred Basmati lines is linked to introgressed QTL (see **Table 1**) and their efficacy under drought stress (**Table 3**). These results confirm that rice lines derived from IR-554190 have a range of mechanisms for sustaining water balance (Venuprasad et al., 2009; Kumar et al., 2014; Dixit et al., 2017). While SBIR-7-18-1 and SBIR-170-258-14 possessed all three introgressed QTL, they were grouped into the less tolerant group based on TDRI, which could be explained by partial introgression of QTL, linkage drag between markers and QTL, or other detrimental QTL. Likewise, the remaining group members (SBIR-16-21-2, SBIR-76-83-9, SBIR-118-104-11, and SBIR-175-369-15) harbored two different QTL in different combinations. Their performance suggests the role of some other epistatic factor that we did not detect in this study.

## 4 Materials and methods

### 4.1 Plant material and experimental design

**Figure 7:**
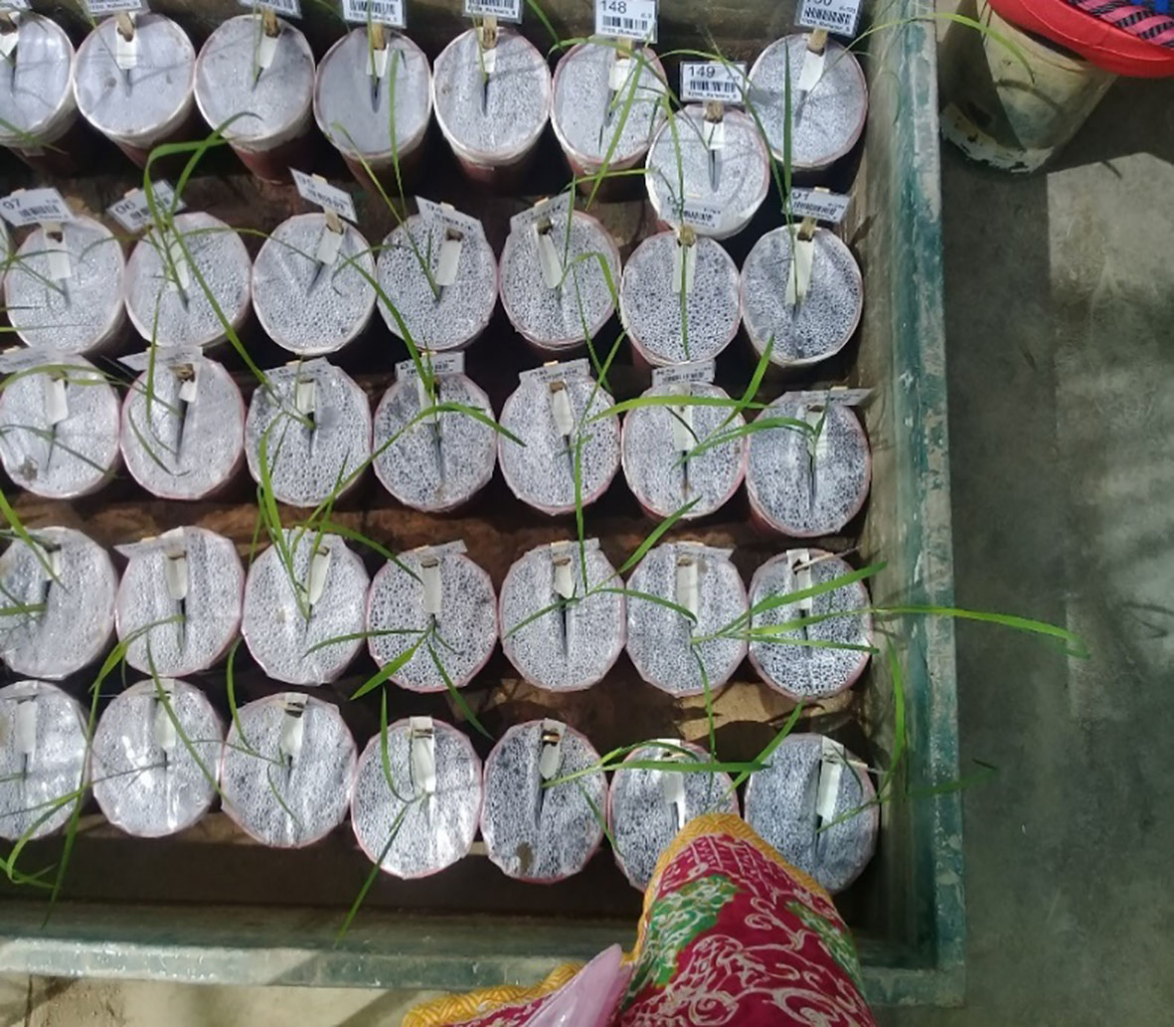
Screening of selected Super Basmati introgressed recombinants under water stress at the vegetative stage in the greenhouse.

**Figure 8:**
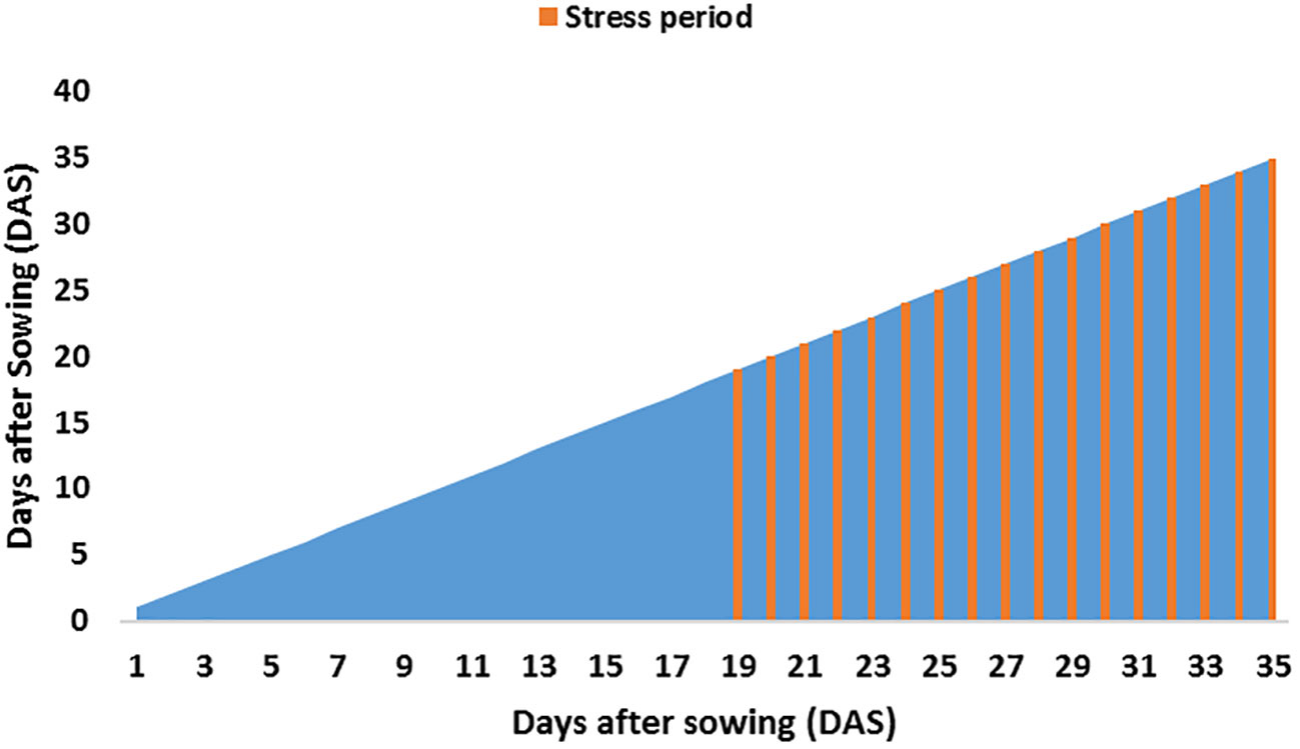
Water stress period imposed on plants.

Eighteen genotypes—15 SBIR lines, two parents [recurrent parent: Super Basmati (drought-sensitive), donor parent: IR554190-04 (drought-tolerant)] and Azucena (drought-tolerant check)—were evaluated for root and physio-morphological traits in a greenhouse at the International Rice Research Institute, Los Banos, Philippines (14°11’ N 121°15’ E, 21m above sea level) during the 2017 dry season (February–April; **Figure 7**). The pot experiment had two treatments [water-stressed (WS) and well-watered (WW)] with two replicates per treatment. Pots were lined with a paper towel and filled with 1.1 kg dried homogenized upland farm soil (**Table 5**, **Figure 7**). Each replicate pot was placed into four shallow open-topped metal tanks (430 cm long, 91 cm wide, 30 cm high) in a randomized complete block design (**Figure 7**). Water was added to each tank to saturate the pots for two days before sowing four seeds of each line directly on the soil surface, covered with a thin soil layer. The water level of each tank was maintained at 60% of pot height. Plants were thinned to one seedling per hill 14 days after sowing (DAS). At 18 DAS, the WS tanks were drained (**Figure 8**). Each pot in the WS treatment was covered with a polyethylene sheet and allowed to dry. Weeding was done manually, with Diazinon pesticide applied once at the recommended rate to control pests during the experiment. The average daily temperature in the greenhouse was maintained at 29.5°C, with the 61.9% average daily relative humidity recorded each day using a Hobo temperature relative humidity (C) data logger (1996 ONSET, USA).

**Table 5:** Description of soil used in the experiment.

Property	Value
pH	6.3
Organic matter (%)	1.38
Sand (%)	13
Silt (%)	45
Clay (%)	42
Phosphorus (Olsen; mg kg^–1^)	46

### 4.2 Physiological and agronomic evaluation

Leaf green area (LGA) was estimated by imaging the plant in each pot at 21 and 35 DAS with a digital camera (PowerShot G7, Canon). Images were analyzed using ImageJ software (ImageJ 1.43; Java 1.6.0_10; Wayne Rasband, Bethesda, MD, USA), with LGA (cm^2^) determined according to the number of green pixels detected relative to the number of blue pixels detected from a 100 cm^2^ calibration card included in the image. The relative growth rate (RGR) between 21 and 35 DAS was calculated as follows:


[ln(LGA from image 2)–ln(LGA from image 1)]/(date of image 2−date of image 1).

At the end of the experiment (35 DAS), tiller number (TN) was counted manually, and plant height (PH; cm) was recorded from the soil base to the plant tip.

A digital chlorophyll content meter (CCM-200 plus, Apogee Instruments, Inc, USA) was used to obtain chlorophyll concentration (CC) in absolute units of µmol chlorophyll per square meter of plant leaf area (µmol m^–2^) on the three youngest, fully expanded leaves on the upper, middle, and lower sections of the leaf on either side of the midrib at 28 DAS and averaged for each plant.

Plant water uptake (WU; kg) in the WS treatment was calculated as the difference in pot weights at the last irrigation (18 DAS) and 35 DAS. Water use efficiency (WUE; g L^–1^) of each genotype was estimated as shoot dry weight (SDW; g) divided by total WU.

For leaf osmotic potential (LOP), one leaf per plant was collected at 30 DAS, stored in a syringe at –15°C, then thawed and pressed to collect sap for osmolarity measurement using an osmometer (Wescor, Logan UT).

Aboveground biomass was determined after harvesting plants at the soil surface at the end of the experiment (35 DAS). The harvested shoots were dried at 70°C for 72 h and weighed (including the expressed leaves used for the LOP measurement) to determine SDW. The percent reduction in SDW (%R_SDW) was calculated as:


[(SDW in the control–SDW under water stress)/SDW in the control]×100

At 35 DAS, roots were washed carefully to remove soil and then stored in plastic bags containing 25% ethanol at 4°C. Crown root number (CRN) and crown roots per tiller (CRPT) were counted manually, and maximum root length (MRL) was measured from the root: shoot junction to the tip of the longest nodal root. For each plant, three nodal roots and their associated lateral roots were scanned (EPSON V700), with the scanned images analyzed using WinRHIZO software (Régent Instruments, Canada) for root length (RL), root average diameter (RAD), number of root tips (RTi), number of root forks (RF) and percent lateral roots (%LR). After scanning, the roots from each plant (including the three nodal roots) were dried at 70°C for 3 days to determine root dry weight (RDW; g). The root: shoot dry weight ratio (RSR) of each plant was determined by dividing RDW by SDW.

### 4.3 Statistical analysis

The mean values from the two replications were calculated for statistical analysis. ANOVA using the least significant difference (LSD) *post-hoc* test at the p< 0.05 significant value was performed for all traits using Statistical Analysis for Agriculture Research (STAR) software of IRRI (http://bbi.irri.org/). Differences between the WW and WS treatments for all traits were reflected in the total drought response index (TDRI), computed as described by Singh et al. (Singh et al., 2017):


(1) $$\begin{array}{l} TDRI={\text{TN}}_{\text{ws}}/{\text{TN}}_{\text{ww}}+{\text{PH}}_{\text{ws}}/{\text{PH}}_{\text{ww}}+{\text{CC}}_{\text{ws}}/{\text{CC}}_{\text{ww}}+{\text{LOP}}_{\text{ws}}/{\text{LOP}}_{\text{ww}}+\\ {\text{LGA}}_{\text{ws}}/{\text{LGA}}_{\text{ww}}+{\text{RGR}}_{\text{ws}}/{\text{RGR}}_{\text{ww}}+{\text{SDW}}_{\text{ws}}/{\text{SDW}}_{\text{ww}}+{\text{RDW}}_{\text{ws}}/{\text{RDW}}_{\text{ww}}+\\ {\text{RSR}}_{\text{ws}}/{\text{RSR}}_{\text{ww}}+{\text{RL}}_{\text{ws}}/{\text{RL}}_{\text{ww}}+{\text{CRN}}_{\text{ws}}/{\text{CRN}}_{\text{ww}}+{\text{CRPT}}_{\text{ws}}/{\text{CRPT}}_{\text{ww}}+\\ {\text{MRL}}_{\text{ws}}/{\text{MRL}}_{\text{ww}}+{\text{RAD}}_{\text{ws}}/{\text{RAD}}_{\text{ww}}+{\text{RTi}}_{\text{ws}}/{\text{RTi}}_{\text{ww}}+{\text{RF}}_{\text{ws}}/{\text{RF}}_{\text{ww}}+\%{\text{LR}}_{\text{ws}}/\%{\text{LR}}_{\text{ww}} \end{array}$$

Phenotypic correlation coefficients among traits were calculated using the mean values in R v.1.0.136, with the graphical matrix generated using the ‘corrplot’ package (https://cran.r-project.org/web/packages/corrplot/index.html).

## 5 Conclusions

Promising drought-tolerant lines had an improved ability to maintain their physiological functions under drought stress. Several lines were superior to both parents and the drought-tolerant check, Azucena. Drought stress significantly reduced TN, LGA, RGR, LOP, CC, SDW, CRN, PH (except SBIR-118-104-11), RDW (except SBIR-62-79-8), RSR (except SBIR-62-79-8), and CRN (except SBIR-62-79-8) and increased RL, MRL, RF, and %LR (except SBIR-62-79-8, SBIR-17-21-3). A strong negative relationship existed between WUE and percent reduction in SDW and root parameters (CRN, CRPT). Based on the TDRI, we identified six genotypes as the most drought tolerant, three as moderately tolerant, and six as the least drought tolerant. The higher drought tolerance abilities of SBIR-153-146-13, SBIR-127-105-12, and SBIR-62-79-8 were associated with greater WUE, maximum retention of chlorophyll content, and maintenance of SDW, RDW, root: shoot dry weight ratio, and crown root number and CRPT with longer roots under drought than the other recombinants. The identified drought-tolerant lines should be further tested and recommended as improved Basmati varieties.

## Data availability statement

The raw data supporting the conclusions of this article will be made available by the authors, without undue reservation.

## Author contributions

Conceptualization: RW and MA. Methodology, investigation, writing-original draft preparation and project administration: RW. Software: RW and FD. Validation: RW, FD and MA. Formal analysis: FZ. Resources, visualization and supervision: MA. Data curation: KS. Writing—review and editing: FZ, AM, MN, MAA and KS. Funding acquisition: FZ and KS. All authors have read and agreed to the published version of the manuscript.
